# Proceedings of the Columbus, O., Academy of Medicine

**Published:** 1877-09

**Authors:** George S. Stein

**Affiliations:** Secretary


### PROCEEDINGS OF THE COLUMBUS, O., ACADEMY OF MEDICINE.

Reported by GEORGE S. STEIN, M.D., Secretary.

Stated Meeting, May 25, 1877.

Dr. Neil, Vice-President, in the Chair.

*Medico-Legal Case.*—Dr. Loving reported the following case:

A physician, a friend of his, had been indicted for selling whisky, it being claimed that the whisky produced delirium and death. The man died on the third day. The patient was under the care of a physician, who gave him thirty-grain doses of hydrate of chloral every two or three hours, until he had taken, in all, two hundred and ten grains. He took two hundred and ten grains of chloral, and one grain of morphia, in twelve hours.

He said the patient was drunk, delirious, and had convulsions; he took these narcotics, and died after taking the last dose of chloral.

What killed him? What was tne cause of death? Was it whisky, chloral, or laceration of the septum between the ventricles of the heart? Which was found after death?

He was inclined to believe that the man's death was produced by the narcotics, and that a laceration of the septum occurred just before death, the heart being distended with blood, and in its violent movements the septum was lacerated.

*Case of Uræmia.*—Dr. Oleson reported a case of uræmia, following scarlet fever, for which he had prescribed hard-cider and muriated tincture of iron; but convulsions came on frequently. He then thought of Dr. Pooley's experience with the old-fashioned corn-sweat, in a case of uræmic convulsions, which he reported to the Academy a few weeks ago.

He tried it, and in a half-hour the patient got easy, and improved. He said that the rapidity with which his patient got to sweating, was interesting to him.

Dr. Loving said that the corn-sweat is fully equal to any vapor-bath that could be devised. He then spoke of the novelty of the thing, and said that if we were in New York, and commenced putting hot corn into the bed of a patient, they would look upon us as outside barbarians.

*Case of Prolapse of the Vagina and Uterus.*—Dr. Kroesen then read the history of the following case:

Mrs. D., aged thirty-nine, married, has had six children; has never aborted; is about the medium height; has enjoyed good health generally until within the last two years; during this period has complained of more or less "weakness" in the pelvic region; menses have been regular until within the last four months; complains of a dragging sensation in the pelvis, with but little pain.

Upon examination, it was found that the uterus was pretty well down in the vaginal canal, and the os pressing upon the floor of the pelvis, its body somewhat enlarged; no heat or tenderness of the vagina; no rectal or vesical irritation; no pain in the back or loins; but there was a certain amount of pelvic uneasiness on attempting to walk, with considerable leucorrhœa. He thought, at first, that it was a case of simple prolapsus uteri, and suspected the possibility of pregnancy; but none of the symptoms of that condition being present, and not wishing to catch up a hasty diagnosis, he left the patient with some directions as to local and general tonic treatment.

One evening he was called hastily to see the case, and found that there was a discharge of water from the uterus, and that she was pregnant, and that an obstruction was about to supervene. The following night was called again to see her, and found her complaining of something protruding from the vagina. On examination, he found a large tumor protruding, apparently as large as a new-born infant's head at full term. It was of a pear shape, the stem end being in the vagina.

A tense congested state of the tumor, with a smooth and tough exterior, was transmitted to the touch. The large end, or base of the tumor, was indented and curved upon itself, and from this part protruded the lower half of a small fœtus.

There was no pain complained of by the patient; no expulsive action of the uterus—only a sense of fullness and throbbing, with discomfort, existed. Physical exploration revealed the uterus to be within the labia, the os enclosing the head of the fœtus with a vice-like grip. An attempt to relieve the head failed, from the firm, constricted condition of the os that held it.

The diagnosis of the case was abortion, with prolapse of the vagina and uterus.

*Treatment.*—Not being able to relieve the fœtal head, the fœtus was returned, together with the prolapsed vagina, within the labia. This was not attained without some trouble in the tumor, because in a measure strangulated. After it was returned the prolapsed organs failed to remain in situ unless supported by the hand. This support had to be constantly given, until the fibres of the os relaxed sufficiently to enable the fœtal head to escape from its embrace. The hips were elevated and the usual treatment given for such conditions; after the cessation of the lochia strong astringent washes for the vagina were prescribed, with general tonic treatment, diet, regimen, etc. The result was good, and the patient recovered without any further trouble.

The cause of this condition was, in his mind, due to a gradual loss of tone in the vaginal walls and subjacent structures, pregnancy furnishing the exciting cause. He thought that during the period that elapsed from her last pregnancy (eight years) a variety of changes in the structure of the parts occurred. The absorption of surrounding tissue and kindred changes the natural strength and tonicity of the vaginal canal might furnish the predisposing cause. The development of the canal and increased weight of the uterus come from the pregnant condition. The enfeebled condition of the sphincter muscles might unite in producing this condition. The occurrence of absorption, uterine and abdominal contractions forcing the abdominal viscera down the canal, might also produce this state of affairs.

---

STATED MEETING, June 1, 1877.

Dr. Neil, Vice-President, in the chair.

*Case of Hemiplegia.*—Dr. Norman presented a pathological specimen of a brain removed from a man sixty years of age, who became hemiplegic three years ago. No complete history of the case could be obtained, except that he had lapses of consciousness—severe pain in the head. A year ago he could not speak or swallow; three weeks ago was struck on the side of his head; no fracture of the skull, but considerable ecchymosis, at the place of injury. Post-mortem showed some softening of the right crus cerebri; some atheromatous degeneration of the arteries; all the arteries of any size were plugged with thrombi.

*Case of Abortion.*—Dr. Pooley presented an ovum, with its membrane intact, of two months' impregnation. He said three weeks ago an attempt at abortion was made, which was followed by a slight oozing of blood for a week or two. Last week a severe hæmorrhage took place, with great pain. He then removed the ovum entire. It showed several punctures, which, he thought, were made by an instrument; there were also deposits of fibrin, which were evidences of recent inflammation.

*Necrosis of the Carpus and Metacarpus.*—Dr. Pooley reported the following case: Nine months ago the patient fell and sprained his wrist; the joint now is inflexible; great swelling on the dorsum; has pain and fever. Has taken iron and quinine for a long time. There were no sinuses. He made two lateral incisions, and all the bones of the carpus and ends of the metacarpal bones were removed, some of them, piecemeal.

Since the operation the patient has done badly; has no pain or fever, suppuration normal, and pus healthy. He has gradually sunk into a condition of lethargy and coma, preceded by delirium. He has no chills nor sweatings; pulse 92, full and regular; arteries are hardened by calcification. He lies in a semi-conscious state; talks deliriously; his face is flushed, but not hot; refuses to take food. He thought his condition seemed to be that of cerebral anæmia.

*Case of Procidentia Uteri.*—Dr. Loving gave an outline of a case of procidentia uteri, in which pregnancy took place, and which he thought a rare occurrence. The lady was twenty-one years of age; has been married eighteen months. When he first saw her she was pregnant; complained of great pain in the hips; she was unable to walk, and had great difficulty in voiding urine. She objected to an examination, and went on in this condition to her full term. He was in attendance at labor, and she refused again to have the necessary examina-made, until she was well advanced in labor. Immediately within the vulva the os uteri presented: the labor went on satisfactorily and she gave birth to the child. Everything went on well after the labor, except that the uterus did not return to its place.

Sometime after this the patient went to Illinois to consult a gynæcologist for treatment, but the last time he had heard of her she was hardly able to walk. He also remarked that this lady suffered with procidentia uteri long before her marriage, and he considered it an uncommon case.

*Laceration of Perinœum.*—Dr. Loving said that modern teachers in obstetrics urge the propriety of attending immediately to laceration of the perinæum. In his time he was taught to wait until after the labor; until the wound had healed over. He had a case once which he stitched, he put in three stitches. His teacher advised him to take them out, which he did.

Two weeks ago he was called to attend a lady in labor—primipara of a rigid fiber—tense habit; at the last pain laceration of the perinæum occurred, not involving the rectum. He immediately put in three stitches, which were removed on the fifth day, the laceration being entirely healed. This he considered exactly the thing to do, and to do it at once.

Drs. Neil and Loving, delegates to the American Medical Association, then gave an abstract of the proceedings and workings of the different sections of the twenty-eighth annual meeting of the Association, held lately in Chicago, Illinois, for which a vote of thanks was tendered them by the Academy.

---

STATED MEETING, June 15, 1877.

Dr. Neil, Vice-President, in the chair.

*Fracture of the Clavicle.*—Dr. Kroesen reported the case of a child, nine years of age, who fell from a wagon to the ground; the shock of the fall was received on the back, producing transverse fracture of the clavicle on the opposite side of the shoulder.

Dr. Pooley said this was an interesting case, and one of unusual occurrence. He thought the fracture was produced by violent contraction of the muscles of the back, and that it could only be accounted for in this way.

Drs. White and Loving also said the fracture in Dr. Kroesen's case could only be accounted for by muscular contraction.

*Cholera Infantum.*—Dr. Loving then inquired of the members of the Academy whether cholera infantum and acute inflammatory diseases of the alimentary canal were not prevailing more than usual in the city at this time, and what was their experience with it? He thought it rather early in the season for these diseases, but he has observed that the first warm weather in June generally brings cholera infantum, cholera morbus, and acute diarrhœa, and that if the weather continues very warm these diseases will continue through July and August; if, however, it gets cool, dysentery will appear, and if it changes again to a warmer temperature, cholera infantum and cholera morbus come on again. He said if this be true, we can, to a certain extent, prevent and avoid them, and prepare and regulate ourselves for them.

Drs. Nunnemaker, Kroesen, Neil, White, and Stein, then related their experience with these diseases.

Dr. Nunnemaker inquired of Dr. Loving whether wet weather had any influence on cholera infantum, and how would he explain it?

Dr. Loving said he did not know if moisture had any influence; if it had, he had no explanation; but he had observed that the temperature of the weather produced the changes which he alluded to. He also observed that in wet, cold summers dysentery is more common than in dry summers.

Dr. Fullerton wished to know whether the treatment could not be improved; the great cause was *heat*, and if we could get command of the temperature, we would have better results in our treatment. If we could devise some apparatus so as to regulate the temperature of rooms, he thought it would influence very favorably this disease.

Dr. Pooley said his experience with cholera infantum was, that if the weather became very warm, suddenly in June, it produced cholera infantum, and also that he was always more solicitous about those cases than with those that occured later in the season.

In regard to treatment two things were important. The first was that concerning the subnitrate of bismuth. If this agent was rightly used, it was a very valuable remedy, and if not, it was one of the most valueless. In small doses of five or six grains, it was of but very little value, but in half-drachm doses it was an admirable remedy. He then related the following case: "Was called to see an infant in the evening, three months old; it looked just as if it was dying; vomiting and purging incessantly; had all the symptoms of collapse; large discharges, of rice-water character; ordered half-drachm doses of subnitrate of bismuth to be given every hour.

The result was, in the morning the child was almost well, the vomiting and purging had ceased.

In older children larger doses must be given, and the remedy must be pushed to do good. If the bismuth does no good in twelve hours we must resort to some other remedy.

The second thing to do is to use cold water compresses to the adbomen. The child should lie on a hard bed, be lightly covered, and the abdomen covered with cold compresses.

This relieves the pain and heat of the abdomen. He also remarked that there was something remarkable in the slight changing of location. Sailing down a river, or visiting some island, or going half a mile into the country had a very marked influence over the disease. When we have severe and threatening symptoms; when the child is exceedingly emaciated, and after the acute symptoms have passed, then he would recommend salicine, one grain every two or three hours, and to anoint the whole body with cod-liver oil.

Dr. Kroesen spoke of the influence of locality, and agreed with Dr. Pooley's remarks. He thought it had a great influence over this as well as some other diseases.

Dr. Loving said that in the use of bismuth we must recollect that it is sometimes adulterated with arsenic, and the large doses might prove injurious. It was also a question with some whether the beneficial effects were not due to the arsenic rather than to the bismuth. He also had observed that after the administration of bismuth the breath had an alliaceous odor, and that it could generally be observed in a few hours after taking bismuth. Another important thing in the acute stage of cholera infantum was the administration of cold water. The almost incessant craving for water was, to him, a most painful symptom. He had labored under the delusion that it aggravated the vomiting and the disease. He now allows his little patients to drench themselves with water; he gives them as much as they will drink.

*Ohio State Medical Society.*—Dr. Pooley then, at the request of the Academy, gave a short sketch of the proceedings of the Ohio State Medical Society held during the week at Put-in-Bay. He said the meeting was not large but pleasant, everything was pleasant, agreeable, and harmonious. There was not a breath of ethics, etiquette, or professional honor. The Society will meet in Columbus on the third Tuesday in May, 1878.

---

STATED MEETING, June 22, 1877.

Dr. P. M. Wagenhals, President, in the chair.

*Case of Iodism.*—Dr. Oleson reported a case of iodism, in which five doses of iodide potassium, five grains each, produced this result. It was given for an ordinary eruption on the leg. The symptoms were lachrymation, swelling of the eye-lids, coryza, and an acne eruption over the body.

Dr. Loving inquired whether the patient had taken iodine or mercureals before, or whether it was not due to an idiosyncrasy. He referred to a case of arsenical poisoning, which was produced by taking three drops of Fowler's solution.

Dr. Oleson said she had taken no medicine for fifteen months.

Dr. Pooley thought this an interesting and important case. Some persons can take no iodine at all, others can take one hundred grains of iodide of potassium at a dose. It is no more likely to produce iodism in large doses than small ones. A good plan is to commence with small doses and increasing them rapidly. In syphilis there is a point of tolerance, and when this point is reached the symptoms disappear.

*Case of Poisoning.*—Dr. Loving reported briefly a case of poisoning with rhus toxicodendron. He said that a solution of iodide of potassium will relieve it, but from his experience he found that

| | | |
|---|---|---|
| | Sodæ sulphite, . . . . . . | ʒii. |
| | Chloral hydrate, . . . . . | ʒi. |
| | Aqua, . . . . . . . | Oj. |
| M. | For a lotion. | |

a much better remedy. It relieves in a very few hours.

*Chronic Bright's Disease.*—Dr. Norman reported the following case: Mr. ———, aged forty years, two years ago had typhoid fever. A week ago, in the evening, was taken with a chill, followed with fever, pulse 120, temperature 103½. Supposing it to be an ordinary intermittent, prescribed a cathartic and thirty grains of sulphate of cinchonidia. Next morning better—next day had suppression of urine, and the next morning symptoms of uræmic poisoning. A tablespoonful of urine was obtained by the introduction of a catheter, and upon examination it was found to be highly albuminous. The symptoms soon were double vision, delirium, contracted pupils. He grew gradually worse and died.

Post-mortem: Kidneys much enlarged with chronic inflammation. The patient died from chronic Bright's disease.

Dr. Pooley said we should never prescribe for patients who complain of severe and persistent cephalalgia until we first examine the urine. This should be the rule of our lives. He also thought Bright's disease an unfortunate name.

Dr. Loving said, a few hours before death several things in this case were important and interesting to him. What was the cause of the extreme itching of the skin? What was the cause of the extreme contraction of the pupils, and what was the cause of the extreme jaundice of the body? The liver showed no disease, and the patient had taken no morphine for twenty-four hours. Could acute or chronic inflammation of the kidney cause these symptoms?

*Cancer of Stomach and Liver—Case of Acute Peritonitis.*—Dr. Frankenberg presented a pathological specimen of cancer of the stomach and liver, removed from a patient who died in St. Francis Hospital. The entire surface of the stomach was ulcerated, involving the spleen to some extent. The liver was full of nodules. He also presented the kidney of a patient who died of acute peritonitis. The abdomen was very much distended, pus in the abdominal cavity, the peritoneum was inflamed and injected. The appearance of the kidneys was that of what is called the "white kidney." On section it showed fatty degeneration. He also stated that the patient had peculiarities, there being four or five ounces of serum in the pericardium.

Dr. Pooley said the specimens were interesting and instructive. The death of the patient from acute peritonitis—it was not suspected at all—but the post-mortem showed marked peritonitis. The patient died suddenly; he had neither pain nor tenderness. He stated that this is the condition in some cases, especially in those of chronic kidney disease. The peritonitis is provoked by the kidney disease. In such cases, we should be on the look out, especially if there is albuminous urine. He stated that the thermometer might have told what was going on. The suddenness of death was an interesting fact. He thought that a splanchnic shock was the explanation of the sudden death,

In regard to the cancer specimen, he said that there was no persistent vomiting as there generally is in cancer of the stomach alone, and that this was generally so in cases of cancer of stomach and liver. The occurrence of cancer of the stomach and liver he thought a rare event. He then raised the question as to which was the primary lesion.

The subject was further discussed by Drs. Pooley, Reed, Frankenberg, Neil, and Stein.

STATED MEETING, July 6, 1877.

Dr. P. M. Wagenhals, President, in the chair.

*Prophylaxis of Cholera Infantum.*—Dr. Oleson read a paper on this subject. He gave a short sketch of the disease—stated that it carried off seven-ninths of the infants in the four hot months of the year. The first prophylactic was proper alimentation; the second, protection from excessive heat; and the third prevention of dental irritation.

[The paper was referred to the Editor of the Ohio Medical and Surgical Journal for publication, and appears in this number.]

Dr. Kroesen reported several cases of cholera infantum. He thought improper alimentation the great cause in these cases.

Dr. Loving thought the cause in the main was the hot weather. The disease generally prevails from the middle of June to the middle of September, and it could only be explained on the supposition of the excessive heat. Heat is the main factor—food and dental irritation are secondary causes. He corroborated every word that was said in favor of the bromide of potassium in the paper.

Dr. Oleson said, as mothers were in the habit of giving their infants soothing syrups and cordials and a variety of domestic teas, that it was a far better practice to give them bromide of potassium. He also believed in dental irritation as a cause, and had relieved many children by the practice advocated in his paper.

The subject of proper food for infants, in connection with cholera infantum, was further discussed by Drs. Frankenberg, Oleson, Loving, and Wagenhals.

